# A study of the provision of hospital based dental General Anaesthetic services for children in the North West of England: Part 2 - the views and experience of families and dentists regarding service needs, treatment and prevention

**DOI:** 10.1186/s12903-015-0029-3

**Published:** 2015-04-09

**Authors:** Michaela Goodwin, Iain A Pretty, Caroline Sanders

**Affiliations:** The Dental Health Unit School of Dentistry, The University of Manchester, Williams House, Manchester Science Park, Manchester, M15 6SE UK; Centre for Primary Care, The University of Manchester, Williamson Building, Oxford Road, Manchester, M13 9PL UK

## Abstract

**Background:**

Patterns of service delivery and the organisation of Dental General Anaesthesia (DGA) have been found to differ across hospitals. This paper reports on qualitative research aimed to understand the impact of such variation by exploring views and experiences of families receiving care in different hospital sites, as well as dentists involved in referral and delivery of care.

**Method:**

Qualitative semi-structured interviews were conducted with 26 people comprising parents (n = 15), dentists working in primary care (n = 6) and operating dentists (n = 5) in relation to DGA. Participants were recruited from areas across the North West of England to ensure a variety referral and treatment experiences were captured. Field notes were made during visits to all settings included in the study and explored alongside interview transcripts to elicit key themes.

**Results:**

A variety of positive and negative impacts on children and parents throughout the referral process and operation day were apparent. Key themes established were clustered around three key topics:

1. Organisational and professional concerns regarding referrals, delivery of treatment and prevention.
2. The role of hospital environment and routine on the emotional experiences of children.
3. The influence of the wider social context on dental health.

**Conclusion:**

These findings suggest the need and perceived value of: tailored services for children (such as play specialists) and improved information, such as clear guidance regarding wait times and what is to be expected on the day of the procedure. These features were viewed to be helpful in alleviating the stress and anxiety often associated with DGA. While some elements will always be restricted in part to the hospital setting in which they occur, there are several aspects where best practice could be shared amongst hospitals and, where issues such as wait times have been acknowledged, alternative pathways can be explored in order to address areas which can impact negatively on children.

**Electronic supplementary material:**

The online version of this article (doi:10.1186/s12903-015-0029-3) contains supplementary material, which is available to authorized users.

## Background

Extensive dental decay in young children and subsequent referral for a General Anaesthetic (GA) extraction is a troubling issue within the UK given the numbers referred and the varying wait times that exist for this procedure [1,2]. Following the requirement that all dental general anaesthesia (DGA) be performed in a hospital setting [3], DGA services were established throughout England. Although there are general guidelines as to the suitability of a child for DGA and clinical guidelines for the use of general anaesthetic in paediatric dentistry [4], these services have developed independently of such guidelines and present differing offers to patients. This could be for a variety of reasons; different workforce, for example, Consultants (in either paediatric, special care or oral surgery), Community Dental Service and General Dental Practitioners (GDPs) work within the service with different approaches and commissioners may have procured certain aspects of particular services. Hospital restrictions (such as estate provision and theatre allocation) could also impact on development, additionally the population served could also have dictated provision based on demand. Previous research has indicated there is a variation in the organisation of DGA services, for example, some services have an “absence of agreed referral protocols and lack of, or unstructured approach to, pre-operative treatment planning consultation and assessment” [2].

Part 1 of this research [1] highlighted the background to this area and showed statistically significant differences in the provision of DGA between multiple hospital sites in the North West of England. It is important to note while broad data collection from referral notes, consultations, observational notes and questionnaires offer an overview of services, they cannot provide the impressions of parents and children, and of those working within and referring into this service. Therefore, while quantitative data can give us an indication of the different services, qualitative analysis is vital in understanding the actual impact on individuals and the difficulties facing those who run these operations in instigating changes [5]. This current paper draws on qualitative research to understand the impact of these differences on service users and their families. In this way this research looks to address a number of aspects of quality as defined by Maxwell [6]. These are ‘acceptability’ how considerately the service is delivered and what the patients think of it, ‘access’ can patients access the treatment when they need it what are the waiting lists and wait times and ‘relevance’ is the service the best that could be achieved taking into account both the needs and wants of the population. These are important aspects of a quality framework within healthcare. Other aspects such as efficiency were not taken into account, as a separate health economic piece would be required for this type of evaluation of quality. Indeed it has been argued that consumers are only able to comment on a set of quality dimensions as laid out by Maxwell [6,7].

Previous research has suggested parents generally view DGA as an accepted form of treatment, with a positive impact on their child, given the reduction of pain and ability to interact socially soon after the operation [8]. However, qualitative analysis has not been applied to the examination of the differences between services or to explore whether anything, beyond the removal of dental decay, could positively (or negatively) impact on the parent or child. This includes their viewpoint on the treatment and operation day, and their subsequent perspectives on dental health and service provision. Additionally, it is important to consider the opinions of the dentists who refer children into these services and those who carry out these procedures.

This study was therefore a qualitative exploration of the experiences and opinions on the service delivery and organisation of children’s dental treatment from the perspective of parents and dental staff connected to the DGA service.

## Methods

Participants were recruited by MG from what could be considered three different groups (see Tables 1 and 2). Firstly, parents of children referred and if possible the children themselves were recruited from 3 distinct settings (although not all children necessarily received DGA extraction as some were treated under an alternative pathway). These sites were part of a larger observational study run across North West England (Table 1). This study involved data collected from both referral notes and questionnaires and was run in parallel to this qualitative element [1]. Secondly, a number of dentists and Consultants who ran the DGA sessions attended by the research team were interviewed; this group also included dentists who ran an alternative pathway, treating child referrals in primary care (without the use of GA) (Table 2). Thirdly, dentists who referred into the system were interviewed following preliminary analysis on the observational data collected on high and low referrers (Table 2). Additionally a commissioner was interviewed who had recently implemented changes to tackle the DGA wait list in an area of the North West England. Parents and children were approached during pre-assessment or treatment planning sessions, in order to determine if they would be willing to take part in the interview. All participants were given the option of a face-to-face interview (either at a community/dental setting, at their home or in another convenient location), or they could participate in an interview over the phone. There was a mixture of participants meeting at home, community settings and some over the phone (although each participant actually met with the researcher at least once). At the start of the interview consent, confidentiality and the purpose of the interview were explained again. Interviews lasted up to 55 minutes.

**Table 1:** **Participant’s details – Parents and children**

**ID**	**Mother/father**	**Child included in interview**	**Area**	**Age range of child at op/treatment (years)**	**Teeth extracted**
259	Mother	Yes	2	5 – 9 years old	1
328	Father	No	2	5 – 9 years old	5
326	Father	No	2	5 – 9 years old	4
648	Mother	No	4	5 – 9 years old	7
901	Mother	No	3	5 – 9 years old	Restoration
920	Mother	Yes	3	5 – 9 years old	Restoration
918	Mother	Yes	3	5 – 9 years old	Restoration
912	Mother	Yes	3	10-14 years old	Restoration
924	Father	Yes	3	5 – 9 years old	Restoration
810	Mother	Yes	1	0 – 4 years old	5
818	Mother	Yes	1	5 – 9 years old	12
811	Mother	No	1	0 – 4 years old	10
806	Mother	Yes	1	0 – 4 years old	4
802	Mother	Yes	1	10-14 years old	2
800	Father	No	1	5 – 9 years old	4

**Table 2:** **Participant’s details - Dentists**

**Dentists treating referred children**			**Referring/non operating dentists**		
**ID**	**Dentist**	**Area**	**ID**	**Dentist**	**Area**
1	Operating dentist	2	6	Referring dentist	1
3	Operating dentist	2	7	Referring dentist	1
13	Primary care dentist	3	8	Referring dentist	1
14	Primary care therapist	3	16	Referring dentist	3
15	Primary care therapist	3	17	Referring dentist	2
			5	Commissioner	1,2,3

Purposive sampling was achieved with respect to the dental professionals by selecting both consultants and dentists involved in DGA delivery, and both high and low referrers. However purposive sampling, which looked to include parents of children of various ages from different geographic areas etc. was not possible as the majority approached were unwilling to take part in an interview. When declining, participants often cited; time difficulties, hectic or chaotic lives, or they were often with a number of children and said it would be difficult to even read through the information at the time let alone find an opportunity to take part in an interview. Parents also seemed uneasy about what they would be asked and it is possible some declined if they were worried they may either be made to feel responsible for the DGA outcome or felt guilt associated with the outcome. A number of parents who agreed to take part when questioned further were actually referred for reasons other than caries, such as teeth needing surgical extraction due to hyperdontia (a condition characterised by having an excess number of teeth). Therefore, we adopted a more pragmatic approach by recruiting opportunistically, and continued with this until saturation of themes had been reached.

Those who agreed to be interviewed signed a consent form and participants came from three different areas of the North West ensuring differences in experience between settings would be captured. Despite the initial problems with recruitment, a wide variation of participants was achieved in the final sample. As data collection was based on an iterative approach, analysis of early interviews guided additional questions in subsequent interviews with initial brief codes constructed and expanded upon as interviews progressed.

Interviews were conducted by MG and included topics such as the parents’ own health, oral health and related day to day activities, challenges associated with stage of development, influences of diet and infant feeding practices. In addition, assessment, treatment planning, prevention clinics, experience at the hospital, previous and future dental visits, and interventions to improve oral health were explored (interview schedule is presented in Additional file 1). Dentists were also asked about treatment planning, prevention their view on the referral process and ultimate DGA and possible interventions which may work for this population.

All interviews were recorded and transcribed, and field notes were taken during (or shortly after) the interviews, these were organised with the aid of the software package Atlas (ti. Version 6.2 2010 Berlin). Thematic analysis was used to identify typical responses and establish the main themes from interviews accounts. Major steps in the analysis process comprised: familiarising oneself with the data, generating initial codes, searching for themes, taking extracts and re-examining and coding in more detail, looking for connections between themes to create new sub-themes in a meaningful way, reviewing themes and defining and naming themes [9]. This type of analysis can also be seen to broadly follow a grounded theory approach which involves open coding, by segmenting the text into labelled sections, axial coding that looks at the relationship between codes and then selective coding by developing the overarching key themes [10]. In addition, while reading through transcripts memos and notes were made on ideas and potential themes that were then explored further throughout the other transcripts. Analysis was therefore conducted by well-developed themes being linked together through accounts of association or connections.

MG also carried out observational work while attending the hospitals on clinic days for recruitment and questionnaire completion. These results have been presented in a connected paper [1] and also prompted additional questions and themes during interviews. It was envisaged that children would be involved in the interview as their voice is an important factor of the DGA experience and previous studies have not usually included this element. This proved very difficult, and although all parents were asked if they would be happy for their child to be involved, the majority declined. They cited various reasons for this including, the age of the child or fear they may be upset talking about the experience. Of those who agreed, the child was often too young to actively take part, or needed heavy prompting by parents to provide even limited answers. Therefore for this work, children’s responses have not been included.

Full ethical approval for this study was obtained from the NRES Committee North West Preston (11/NW/0503) and all parents/guardians gave informed written consent before taking part for themselves and for their child. If children were over 11 years old they were also asked for their permission to consent, in addition to their parents.

## Results

**Figure 1:**
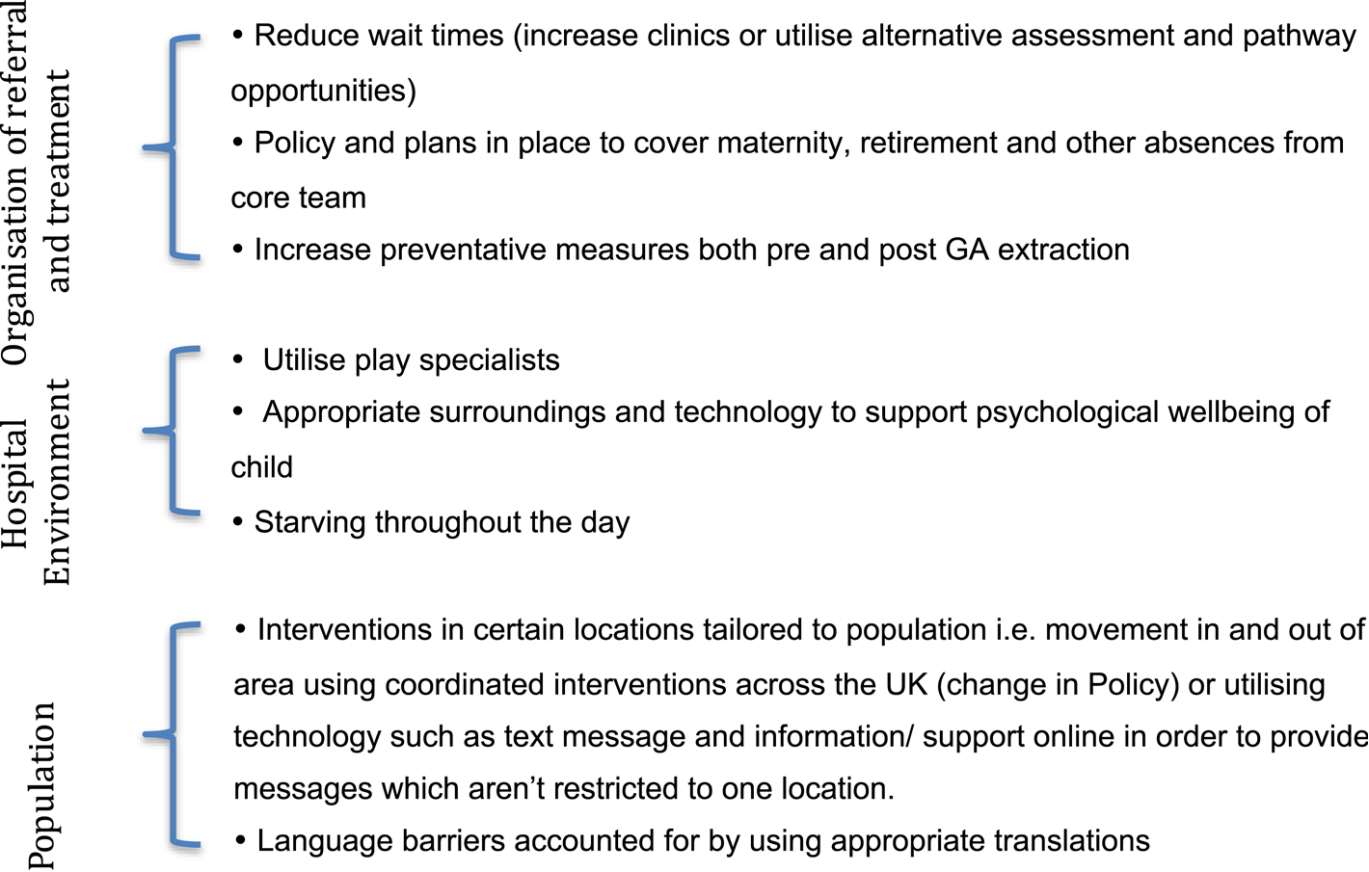
**Quantitative themes established in Part 1.**

Analysis revealed a number of themes running throughout responses from both parent and dental providers. Three overarching themes correspond with issues reflected in the observational and quantitative data collection in part 1 [1]. Part 1 detailed three key areas that differed throughout hospitals and identified aspects within those categories, which could potentially improve or modify processes for the better. These themes from Part 1 are presented in Figure 1.

**Figure 2:**
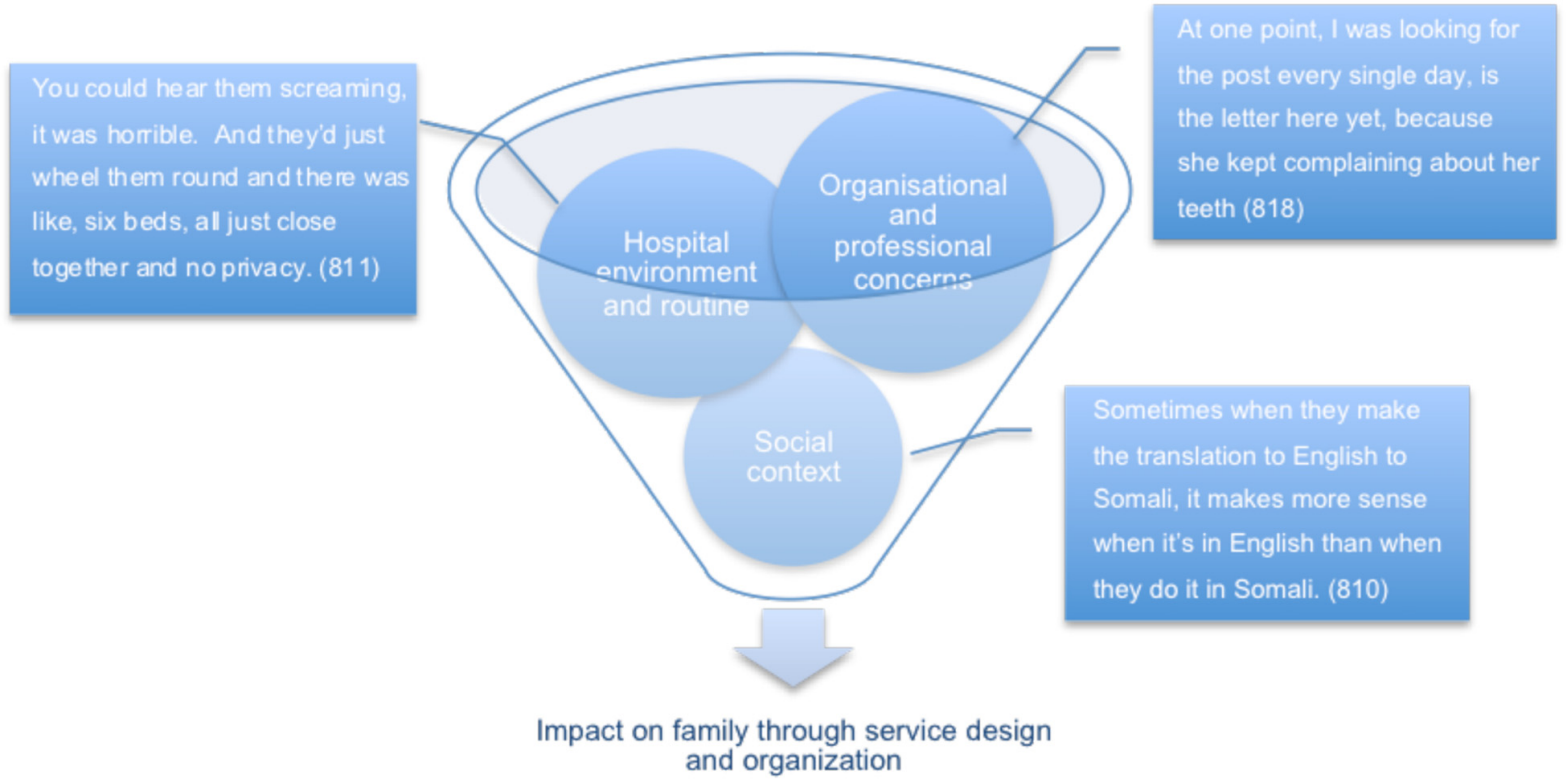
**Main themes established which influence service delivery and organisation.**

The issues described in Figure 1 corresponded to themes that emerged from qualitative analysis, and therefore these key areas determined from the quantitative paper could be expanded upon using qualitative analysis. Each theme will be addressed below with ensuing qualitative quotes, which either corroborate or refute the established themes; Figure 2 illustrates each developed theme with a contributing quote.

### Organisational and professional concerns in referrals, delivery of treatment and prevention

Many respondents discussed the wait time for their child’s DGA. This was both in a positive and negative light. When parents had been advised there would be a long wait they seemed pleasantly surprised their operation was sooner than expected:

*I’m fine with that because at first at the hospital he told me the waiting list would probably be five or six months. So I just had in my mind that it’s going to be Christmas, New Year. So yeah I was really surprised with these appointments actually. (Area 1; Parent 802)*

*That was quite quick I got the initial referral within the first month so that was ok cause I was expecting the referral to be 6 months so I was shocked at that (Area 1; Parent 806)*

However others expressed concern over the wait and the negative effect it had on their child. They not only related the pain and discomfort their child endured during their wait but also on how they felt this was out of their control and all they could do was await the appointment. This was particularly apparent through discussions with parents, to whom dentists intimated they were not able to provide anything additional for the child until the DGA was performed. Several participants expressed their frustration at waiting, particularly when they felt it had directly resulted in pain or infection. This is an important factor, which needs to be fed back into the referral process. While it is not always possible to reduce the wait time immediately, referring dentists must be encouraged to continue treatment for any pain or infection during the wait. This links strongly to payment mechanisms and prevention discussed later. Additional treatment provided outside of the hospital by the regular GDP and discussion of potential wait times could alleviate, to some degree, the frustration and lack of control felt by parents may be alleviated to some degree. As it would allow them to seek alternative treatment if required rather than waiting for the appointment. However, there appears to be a sense that, once referred, the child was no longer the responsibility of the dentist.

*At one point, I was looking for the post every single day, is the letter here yet, because she kept complaining about her teeth, and every time I took her back to my dentist, he was like, well there’s nothing I can do, it’s in the hospital’s hands now, so it was kind of like, well what do you do? (Area 1; Parent 818)*

*We went back to the dentist because it was a long wait …and there was no surgery in sight, he says he's now got a Buccal abscess, he's been complaining he's got a bit of gum soreness (break) It was just shocking service that he gets referred by the dentist in March time, it takes eight months to get it done (Area 1; Parent 800)*

*And he was in real, real pain so we just wanted to try and get this done as soon as possible, so I can’t really remember how that happened, but he had lots and lots of antibiotics and eventually we were sent to (Area 4) (Parent 648)*

Those working in the field were also aware of the wait and the negative effects it could have on children referred. A number of dentists, consultants and commissioners in the area had looked to alternative pathways, involving treatment in primary care, in order to alleviate the wait.

*So many children, young children who were in pain were apparently waiting for months and in fact we were told there were 400 on the list at one point. So therefore something had to be done (Dentist 5)*

Staffing issues were a factor discussed in the connected paper, Part 1 [1] as they affected wait times and had been mentioned as an issue to the authors by members of staff at various hospitals. This was echoed by certain parents who discussed concern over staffing and the effect on the ability to see and treat children in a timely fashion.

*Their trouble was they couldn't find surgeons to do the lists, and therefore they would have the slots to do the lists, because the slots wouldn’t be available at the children's hospital, they'd have no surgeons. And they were just being done on an ad-hoc basis; it was absolutely rubbish (break) they need to be getting their arse into gear and getting more dentists (Area 1; Parent 800)*

#### Reasons for referral

Common reasons for DGA referral include; non-compliance, age, number of teeth needing to be removed [4]. Another aspect outlined by both parents and dentists was the benefit in keeping the regular dentists separate from treatment, which might ether, induce anxiety or cause distress in order to avoid potential issues with dental attendance in the future.

*Because it’s quite beneficial from our side because we’re the nice guys, and then we send you to that place to have teeth out and then you come back to us and it’s all alright [Laughter]. So having that distance is actually quite helpful (Dentist 6)*

*(talking about her dentist referring her child for GA) he said he had children the same age as xxx and xxx despite being a dentist he knew how they felt about going so that way he would rather not do it there (Area 2; Parent 256)*

*No I think he's fine with it and I wanted to keep it that way - I didn’t want him stuck in a chair with someone with a foot up against his head tugging at his teeth with a pair of pliers while he was conscious (Area 2; Parent 326)*

#### Prevention and previous treatment

This section describes prevention and previous treatment given by dentists; a more in-depth analysis of additional interventions is the subject of another paper. Data collection from 456 patients attending hospitals throughout the North West indicated there were differences in the preventative advice and treatment being given by dentists across districts. While the majority mentioned their dentist had discussed the use of adult fluoride toothpaste and low sugar, there were those who stated they had not received this advice. This was reiterated by a small number of parents within the interviews.

*I just think…I think we weren’t told anything about the content of fluoride in toothpaste, at all, at a previous Dentist. We’ve only found that out through going to our recent Dentist, which obviously when he goes to when he’s six, you know, it’s too late by the time he’s six. (Area 4; Parent 648)*

*It’s helpful, you know, when you don’t know anything you just brush, but when they tell you, like, this, and it’s helpful. (Area 3; Parent 918)*

Additionally repeat dental general anaesthetics for either that child or another child in the family was worryingly high throughout participating hospitals but particularly for two in the North West. Several of the parents interviewed commented their child had already experienced a dental general anaesthetic or a sibling had teeth extracted under GA. When the researcher asked if any prevention such as fluoride varnish or fissure sealants had been applied to that child or any other siblings following a previous DGA one parent responded

“*No, none of them have ever had anything like that… he’s never gone over anything like varnishing or anything like that.” (Area 1; Parent 811).*

Fluoride based treatment such as fluoride varnish was also mentioned as an element of pre-emptive treatment which was lacking.

*“The issue really is that I don't think there is a lot of prevention going on in primary care” (Dentist 2).*

Other dentists expressed apprehension over the use of these types of treatment (which might go part way to explaining the infrequent use). There were concerns it did not tackle the primary cause of caries and also unease over the reasons behind it being pushed as a preventative measure especially in comparison to fissure sealants that have a sound evidence base and require less frequent application.

*The rule says you put the fluoride on, so that’s how we prevent problems. Well hang on, why is this? I mean, and I think I was alluding to it, suddenly after Colgate bought Duraphat or what have you, and the Government decided on a preventative agenda that needed to be pushed, (break) So, one strategy involves twenty applications of topical fluoride and the other strategy is a one off fissure seal, which has been shown to be more effective …but why don’t they get us to do that? “Oh, we’d have to give you some UDAs for that”. And it’s unbelievable. The beauty of the UDA system is it would have been very easy to modify (Dentist 16)*

A number of the dentists working in the area of GA extraction commented that repeat GAs were a definite concern. One of the ways to counteract this was to remove not only teeth that cannot be restored but also any teeth that have signs of decay once a child is referred. This would result in a child being returned to a caries free state and given the best opportunity to continue in good oral health.

*One of the things I try and say to the parents is that if there are any other carious teeth then we get rid of them and you start from base again and hopefully the prevention will then kick in from then and we don’t get repeat episodes (Dentist 1)*

*If they go for a general anesthetic, then we tend, once that decision is made, then we tend to be quite radical about it, so what we don’t want to do is leave anything behind with a hole in it which is going to flare up and cause trouble in the future or warrant another general anesthetic (Dentist 3)*

While prevention advice was provided at some hospitals this was not a standard procedure throughout the different locations visited. While few parents discussed prevention from referral to a hospital setting those that did were encouraged by advice and techniques to improve their child’s oral health.

*She’s given her these purple plaque detector things, that you put in your mouth (break). And you can see…it’s like a guide basically, so you can work on the areas that you’ve not been cleaning properly. And she loves it. (Area 1; Parent 818)*

### The role of hospital environment and routine on emotional experiences of children

As stated in Part 1 of this series [1], a family’s experience of GA could differ significantly, partly due to the restrictions of each clinical setting. Observational information collected illustrated the different processes and procedures used. One most frequently mentioned by parents was the communal recovery stage for a particular hospital that impacted on privacy and dignity. The distress caused both to the parent and their child of recovering in a shared area was apparent. Parents reflected on the panic induced in their own child from seeing others in distress and the additional impact on their anxiety and barrier to recovery with this lack of privacy. Dentists also discussed the restrictions of bed space, which also feeds back to the previous topic of wait times.

*They take you to recovery, but they’re not even properly awake, when you go, they’re just screaming. And they’re not private neither, so every time one was coming up, you could see…and it was horrible because one of them was just like ripping wires out and I was like, oh! (break) You could hear them screaming, it was horrible. And they’d just wheel them round and there was like, six beds, all just close together and no privacy. (Area 1; Parent 811)*

*The other children, you can hear them all distressed and stuff like that, and that panics them anyway, and I don’t think it helped in that respect (Area 1; Parent 818)*

*Because we have a limited number of beds and have to go through the whole protocol of being admitted into hospital so it limits the numbers quite dramatically it also takes away that scope of being able to offer urgent appointments (Dentist 1)*

Observational work also noted administration and assessment on the day in each hospital differed. Some clinics asked all parents to arrive at the same time to be assessed by the dentist, anaesthetist and nurse in one period, followed by operations occurring one after the other over a few hours later. Other clinics saw patients individually for their assessment and operation in one slot. Both dentists and parents commented on the long wait for children when they were seen in groups and again the negative effect, particularly for young children.

*They come in at 12.30- but if they were to be seen at 2 o’clock the would have to have been starved so consequently there all starved from 8 o’clock - to keep them waiting till 5 o’clock before they go into theatre particularly if they are only 5,6,7 years olds it’s a long time (Dentist 1)*

*I think they'd be better off making the dental sessions in the morning because then the child's been basically starved for the night, you could wake them up at five to give him some…well, the lists wouldn't start till nine, so he could have some fluids at seven, and he wouldn't have to spend the whole day being starved. I think that's a big thing, was that he starved all day for a four o'clock list. It's much better if you just had the lists in the morning and it's a lot less stressful. (Area 1; Parent 800)*

Despite the issues of starving during the day many of the parents were positive about their experience, the staff at the hospitals and the ultimate outcome. While certain factors such as recovery were a traumatic aspect to the process many parents were optimistic about the treatment as a whole and acknowledged the necessity of going through the procedure. Parents also commented on positives following the operation, resuming essential activities such as being able to eat without choking

*The staff were fantastic, at (hospital 2) I have to say. I mean its a long day as he was the oldest he was the last on the list, but, and it was a hungry day wasn’t it (to child) we were starving, had nothing since 7 o clock that morning and he didn’t have it done until half four (Area 2; Parent 259)*

*And then literally, it was over and done with, within seconds she was under, quite quick, which was fine, and then like one of the nurses came out with me, and she was like, you alright, you okay? (break) She was still bleeding quite a lot when she came out of hospital, and she was very, very docile, and then it was the nightmare of going into the massive multi-storey car park, while she’s still coming out with loads of blood…it wasn’t the nicest of experiences, but having said that, it was necessary. (Area 1; Parent 818)*

*I mean it, as a parent, I, myself, I actually had a terrible time having teeth out when I was younger, so they were absolutely brilliant with me. (child) was fine because I was putting on bravado with him, but as soon as he went to sleep I was in floods of tears, and I have to say the staff were excellent- and they were just brilliant; I couldn’t fault the service there. (Area 4; Parent 648)*

*She’s actually been better, she’s not choking as much on her food for some reason, so it must have been the pain in her teeth that was making her swallow. (Area 1; Parent 811)*

#### Child friendly environment

Given the young age of many of the children referred for DGA, parents were asked specifically about the hospital environment and any positives or negatives that fed into the experience. Again hospitals differed in their approach to involving children. One notable difference in some hospitals was the presence of play specialists. These were individuals brought in specifically to talk children through the procedure and, as commented by one parent, spoke *‘at the right level for kids to understand’ (Area 2: Parent 326)*. Often the play specialist was also available at the pre assessment and therefore continuity was kept with whom the child saw and what they expected on the day. Parents commented these specialists made children feel less anxious and more aware of what the day would entail.

*Certainly the presence of a specialised child specialist play person really in terms of the difference in training to speak at the right level for the kids to understand what’s going on to alleviate the fears can only be a good thing (break) They took (child) round and went through the procedure with them in a very simple way so he could understand it and in a way that wouldn’t induce anxiety (Area 2; Parent 326)*

*She [play specialist] went over absolutely everything that would happen, and she was really good as she was jogging his memory obviously she had spoken to him at the pre op the first time so she was reminding (child) what was going to happen so everything through from putting the cream on their hand, to what was going on their hand and jogging memory and he was remembering what colour it might be and things like that, no she was very good. (Area 2; Parent 259)*

*(At hospital Y)There was no showing what was going to happen to him or explaining that he was going to get put to sleep. At (hospital X) they told him about fairy dust and, you know, all sorts of stuff to make it nice, and they told him about the tooth fairy. They didn’t do any of that at (hospital Y); it was just in, ‘We’re going to sort him out. You go and sit there and we’ll bring him back in’, and that was it, whereas (hospital X) was much, much better. (Area 4; Parent 648)*

Hospitals without a play specialist still often had a child friendly environment with activities for children and, on occasion, entertainers such as clowns who came into the ward. Families readily welcomed all of these aspects of quality provision.

*(Discussing a clown at the ward) They were all looking at him and listening and he was just telling them jokes and everything, everybody was laughing and the children… so it was fun (Area 1; Parent 810)*

### The population and influence of the wider social context on dental health

When looking at any differences in population between hospitals it was apparent hospital 1 had a greater number of languages spoken and a greater proportion of families who had moved since their child had been born. One participant, whose first language was not English, commented on the difficulty of translating materials such as leaflets and the loss of information and context that can occur. As noted in Part 1 [1], differences between DGA services makes distributing information increasingly difficult as, even if translations can be made available, the information given may be more confusing with a greater chance of misunderstanding if translation is difficult for the language in question.

*Sometimes when they make the translation to English to Somali, it makes more sense when it’s in English than when they do it in Somali. (Area 1; Parent 810)*

The migration of people from other countries was also a factor mentioned by dentists causing difficulty in treating caries early. Dentists stated often by the time they first see a patient it can be too late and for these patients who have only recently moved to the area or into the country nothing within this service could have been done to prevent the extent of the decay.

*They're first timers and they’ve come to England. The parents and the children, they're very nice people but a lot of these patients have not been able to access quality dental care and they’ve got issues with health (Dentist 17)*

Parents and dentists, in relation to maintaining good oral health, discussed the realities of day-to-day life. Diet was obviously a significant factor and parents reflected on the difficulties in controlling their child’s diet, particularly when there were a number of children of different ages and other family members who looked after and had influence over diet and oral hygiene. Behaviour is an incredibly complex and difficult area to change. However it is proposed when behaviour that causes the initial GA does not alter, subsequent need for GA extraction could occur. One parent did reflect on their behaviour and the transformation following their first child’s GA, stating a change in the use of a baby bottle at night. However, it appears while one problem was tackled, other contributing factors towards dental decay were not addressed such as brushing and excessive sweets which were identified as the cause of the second DGA within the family. Again once knowledge was gained, this can then prompt a behavioural change by restricting what food was purchased and available in the home.

(Parent (R) speaking about her first child who underwent DGA to Interviewer (I))

- *R: You know, we don’t know anything, we give bottle, and make him sleep and that’s very bad. Very bad.*
- *I: So with your younger children*
- *R: I changed, yes.*
- *I: …So with his (child referred for GA) teeth, what do you think were….*
- *R: I think, brushing…And he eat loads of sweets.*

(When questioned further as to where the challenges are with the child’s diet)

- *R: The shopping. Even, you know, at home, when I’m giving him cereal he has to put sugar on it. This is challenge to me at home. Sometimes, when I’m around I can look after them. When I’m not around they can use sugar, and things, and so now I make, you know…every time when I go to shop I have to buy sweets, sweets, sweets, now, when I’ve seen his teeth, and the dentist told me, this is about the sweet, I have to stop it. Now, I made one day. Only Friday. (Area 3; Parent 918)*

## Discussion

The main aim of this paper was to explore the themes that reflect the topics developed in *Part* 1 of this series generated from observational data collected at hospitals where qualitative interviews took place. Qualitative interviews are able to shed further light on observational data [5] collected by the research team and give both a patient and service perspective on the impact and reason behind differences in the delivery and organisation of services and the population who attends them. While it is acknowledged both patients and dentists will have different perspectives on this area. It was felt it was important to include both within this work. Dental professionals have an important role in both the referral and dental procedure carried out, as they are the gatekeepers and ultimate decision makers in regards to treatment. While parents play an essential role in their child’s health and wellbeing and both children and parents can be affected by their experiences and reaction the procedure.

It appears both parents and clinicians see DGA as a vital service when severe caries is experienced in young children. However certain aspects of individual services stood out as making this experience less traumatic for both parent and child and taking opportunity to reiterate preventative advice and treatment, which could lead to improved oral health in the future (although from this work we cannot know if any behaviour change was effectively implemented or continued for any time). These impacts and improvements clustered into three main themes that are described and expounded on below.

### Organisational and professional concerns regarding referrals, delivery of treatment and prevention

One important issue was the wait time experienced by some individuals resulting in prolonged pain, recurrent abscesses, etc. This negative impact was recognised in a connected paper already published by the authors [11] where an extended delay was associated with increased disturbed sleep and pain experienced thought out the wait. This was also acknowledged by those who worked in the area and was one of the reasons for the establishment of the alternative pathway within one particular region to alleviate the pressure/wait for one particular hospital included in the research and attempt to treat children in an alternative setting without GA (discussed by Dentist 5 within the organisational and professional theme).

The need to be radical with treatment and remove any teeth with poor prognosis was a factor mentioned by dentists in an attempt to avoid a repeat GA. It could be seen within some hospitals a full clearance occurred for some children when decay was so rampant that any teeth left may have resulted in a re-referral. This has been demonstrated in previous research, one paper indicating that 75% of single tooth extractions required repeat DGA for caries left at initial DGA [12]. The paper suggested a radical treatment approach to reduce the need for repeat GAs. Further research has indicated repeat DGAs, for a child and within the family, are still a significant issue [1].

*Part 1* of this series noted the need for effective prevention in primary care in order to reduce the number of children being referred for DGA [1]. However another important aspect is that for some children a dental visit and subsequent referral to a hospital for DGA may be one of their first experiences with a dentist (as discussed in the results section) and the opportunity to not only return them to a state of good oral health but also to instil the importance of maintaining oral health, cannot be missed as every contact with a dental professional should count in relation to prevention. Opportunities to change behaviour are an important aspect of various health psychology interventions and research, i.e. changing risky health behaviours such as smoking, these have been labelled ‘teachable moments’ [13]. These teachable moments describe naturally occurring health events such as cancer diagnosis for smoking cessation whereby perception of personal risk and emotional response can be identified as a time to facilitate change [14].

A study conducted in New York showed those children undergoing DGA who attended a follow up appointment within 2 weeks that went over preventative advice, were less likely to develop new carious lesions than those who failed to attend. However one significant issue with this was that the failure to attend rate was high with only 39% attending the immediate follow up [15]. Additional research has suggested not only a radical approach to extraction but also special preventative care is required as high risk children seem to not respond to regular preventative care and often return for a repeat GA [16]. One hospital had seized this opportunity by having a dedicated prevention clinic, which had to be attended before the child could be seen for their operation. While this is not a definitive solution to correcting poor oral health and associated behaviours, given the variety of complex causal pathways that lead to the need for a DGA, it is an aspect of service delivery that may tackle decay in high-risk children.

Good oral hygiene and behaviour remains an important factor in maintaining oral health however this is a difficult area to change as it is influenced by complex processes including social determinants of health [17]. Preventative measures should also be instigated, if possible, from all health professionals involved in keeping with the NHS strategy of ‘making every contact count’ [18]. A number of Dentists discussed both fluoride varnish and fissure sealants as tools to aid preventing dental caries. One particular dentist questioned why fluoride varnish was promoted and supported via UDA payment over fissure sealants. Fissure sealants have been shown to be superior over fluoride varnish in preventing decay [19] and are particularly relevant for this high risk population as patients are often irregular attenders [20,21], making constant application of fluoride varnish problematic [22,23]. Fissure Sealants have been shown to reduce caries up to 48 months when compared to no sealant [24] and therefore maybe more suitable for a population who attend infrequently, although establishing regular dental attendance should always be the goal of both parent and dentists in regard to high risk children.

### The role of hospital environment and routine on the emotional experiences of children

Parental emotions and experiences and children’s experiences, related by proxy, regarding both the day of the GA operation and recovery period were explored. All parents interviewed felt extraction under GA was the only treatment option for their child whether alternatives such as LA had been tried or not. It was apparent for some the experience of not only the child going under anaesthetic but also recovery was distressing; they were shocked at the amount of blood and by the emotional state of their child. Other children being present during their child’s recovery could have exacerbated this. Parents commented this was upsetting for both themselves and their child. In future it would be advisable to prepare parents and children what they will experience on the day as many of the negative aspects of the service such as wait times were lessened when parents were warned about specific parts of the process. Conversely if parents and children were provided with too much detail on the potential negative aspects of DGA this could increase the fail to attend rate and therefore this is a subject that would have to be handled with delicacy.

The ability to aid not only the physical but psychological wellbeing of a child is an important factor. There is a growing body of literature that emphasises the need for children to have the right to information and participate in the decision process, depending on age and maturity [25-27]. In this respect the play specialists or those who interacted with children and discussed the procedure in a way they could understand were a valuable asset and continuously discussed in a positive light by those interviewed. Work that specifically looks at play specialist involvement such as Hubbuck's book *Play for sick Children: Play Specialists in Hospital and Beyond* note that for most children the hospital environment is entirely abnormal compared to their everyday home life and day-to-day activities’ [28] this book emphasises the importance of the play specialists role within a multidisciplinary team but is more descriptive than results based. Few would disagree that any action, which can calm and prepare children for anaesthesia should be encouraged however there also needs to be more research conducted to confirm the evidence base of this benefit. This engagement and use of play specialists had been incorporated in a number of the hospitals observed within this particular research.

The child friendly nature of certain hospitals was also commented upon in a positive light. In recent years the impact of hospital environments on a child’s emotional wellbeing has been of increased interest and activities for children not only to relieve boredom but also reducing isolation and anxiety have been described as invaluable within a hospital setting [29], this was also reflected in the hospital settings with parents commenting on the activities, technology and organisation of the settings impact on their child.

The majority of parents reported a positive impression of treatment following their child’s DGA. This is in keeping with previous research [8,30] indicating an improvement in quality of life and overall health occurred following treatment under DGA (usually within 2 weeks). It should be noted that even those, in this study, who found the experience distressing acknowledged its necessity and positive outcome for their child.

There were very definite positives and negative aspects to services discussed with both parents and dentists. A number of these were beyond the control of the service given restrictions within the hospital setting. For example, privacy in the recovery area outside the operation theatre was often shared with other children undergoing the same treatment. However aspects such as play specialists (or staff who took time to involve both parent and child) were consistently mentioned as an invaluable aspect to the service.

### The influence of the wider social context on dental health

Success for implementing prevention advice and behaviour change within high-risk children is largely influenced by the social context of the population [31,32]. It is important to note any prevention must acknowledge the diversity of the group and this should be included in the design of preventions to tackle this issue. This includes the ability to appropriately translate material but also address the frequent relocation of parents and children within certain locations in trying to give a consistent message. New technology could be incorporated such as information online and via mobile phone. These types of interventions have started to be used both within oral health education and also other areas connected to oral health using the common risk factor approach such as weight gain and diet advice [33,34].

#### Limitations and future research

The number of interviews conducted and the representativeness of the conclusions generated to the rest of the population limit qualitative findings from this research. Also given the number of parents approached to take part in interviews and those who actually agreed (41% consent for parents 29% for Dentists) means an important part of the population could have been missed. This is a problem with the majority of research; those who don’t consent can often be the people we most want to hear from. However the interviews appear to contain a range of respondents, with children of various ages having a number of teeth extracted (ranging from 1 to 12), both mothers and fathers were included in interviews with participants attending a variety of hospitals resulting in a varied combination of respondents for interviews. Unfortunately the majority of interviews did not include children or children were too young to effectively contribute to interviews alongside their parents. A child’s opinion and view is an important aspect and in the future additional efforts should be made to involve children of an appropriate age to gain their views.

A range of dentists were included in interviews, from those who conduct DGA operations; those who refer in to the service and those who treat referred GA children in a primary care setting. Given the combination of this research alongside a large quantitative sample, this blend of information can be used to enhance the understanding of this potential complex area of service design and delivery. In this way the data complements one another as qualitative research is not usually used to generalise to the wider population but in this situation can be used to better understand the quantitative and observational data acquired and give validity to the conclusions and themes established. In this way the research has not used methods simply to satisfy a tick box approach but to create research which logically allows for the most important and diverse information to be gained [35].

As the female researcher (MG) was from a non-clinical background it was felt this was important to reiterate to both dentists and participants. It was believed this meant that certain topics could be addressed without parents or dentists feeling they were being judged or scrutinised by someone within the profession and that there was a genuine interest in their *individual* views and how they perceived the impact of GA without a predefined assumption of what this should entail. This was discussed as the main focus of these interviews with participants. However, having said this, there is in any interview recognition that information gained is in part directed by the line of questioning and given the research area, parents and to some extent dentists may have felt certain responses were required of them.

Several aspects which anecdotally have shown to be a positive feature when attending for a DGA such as play specialists could be further explored on a wider scale between hospitals to see not only the impact on the day of the operation but on a short and long term basis in relation to anxiety, subsequent hospital or dental visits and recovery following a DGA.

## Conclusion

**Figure 3:**
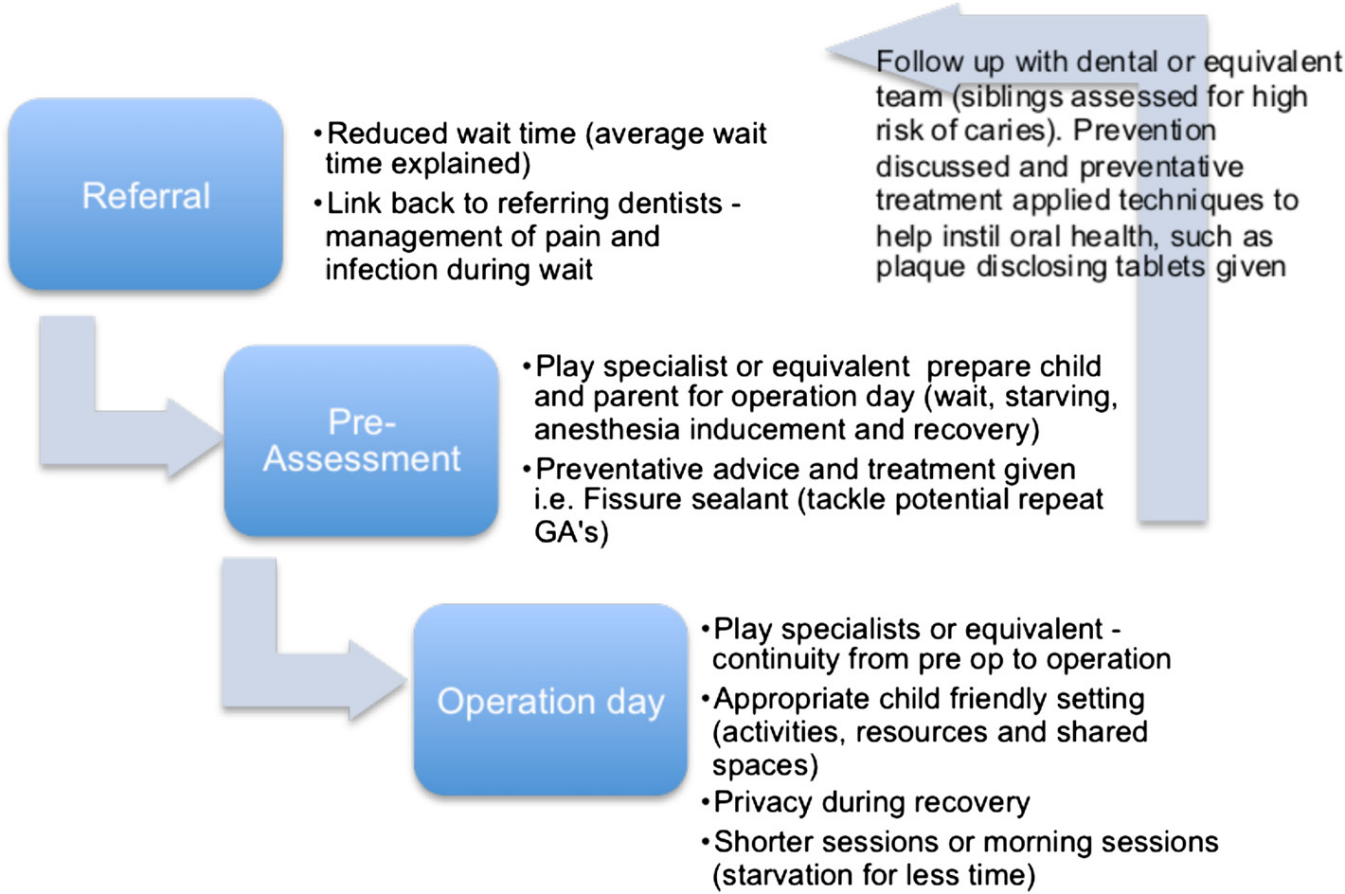
**Best practice – a reference standard DGA service.**

This paper has discussed the effects on families of a paediatric referral and subsequent extraction of carious teeth across a number of sites in the North West England under GA. It is clear the setting and organisation within a hospital can have an impact on the emotional wellbeing of the child and a balance has to be sought between being able to see and treat children in a timely fashion alongside treatment which reflects not only the physical but psychological needs for that child. It is important to consider how these impacts and differences observed can translate into improving patient care and experience. Given the information gained from the quantitative data collected from referral notes/questionnaires, observational data and qualitative interviews, the authors have constructed a ‘reference standard’ DGA service, which incorporates ‘best practice’ from the hospitals visited. While this is not always attainable given restrictions to the layout and structure of hospitals settings, it does give context to the best practices across hospitals that can be implemented throughout DGA services (Figure 3).

## Additional file

Additional file 1:
**Interview schedule: Parents and children.**

## Abbreviations

GA: General Anaesthetic
DGA: Dental General Anaesthetic
GDP: General Dental Practitioner
