# Supplementary material for: A study of the provision of hospital based dental General Anaesthetic services for children in the North West of England: Part 2 - the views and experience of families and dentists regarding service needs, treatment and prevention
Source: BMC Oral Health. 2015 Apr 9;15:47. doi: 10.1186/s12903-015-0029-3 (PMC4407771; doi:10.1186/s12903-015-0029-3)
Supplement: Additional file 1: — Interview schedule: Parents and children. [file 12903_2015_29_MOESM1_ESM.docx]

**Interview schedule: Parents and Children**

Reducing the number of General Anesthetics required for tooth extraction for children in the North West of England - a multi staged approach - Interview Schedule

***Questions relating to oral health and hygiene related to other day-to-day activities***

- Firstly can you just describe your household to me - how many people live there, what ages etc?
- Please describe your day-to-day activities - what is your normal routine with you and your children ... how does oral health fit into this in the morning and evening.
- Are aspects such as getting everyone to brush their teeth in the morning or night a habit or is it something you have to consciously remind yourself to do?
- What are the difficulties or challenges do you face in keeping to a routine?
- Has anyone given any really good advice that you would offer to other parents?
- What behaviors are the biggest challenges for you to try to change for your child and/or your family?

***Questions relating to influences on diet***

- Were/ have you ever been given any information about diet and how food and drink can affect oral health (or any aspects of health) - if yes where did you get this information from (probe- did you believe it/ did it make you change your habits
- Thinking back to your first child what were your views and opinions on food did these change throughout your child growing older (if yes how)
- Are there any types of food or drink that you know can affect your/ your child’s teeth- were you aware of the impact of these things in your child’s diet early on - where did you get information from
- Do you ever discuss diet with other friends and family - what impact if any do you think this has
- Many parents report that their kids put pressure on them to buy specific foods. What kinds of foods do your kids ask you to buy that you are not sure you should?- How do you deal with these requests?
- Does your school have any policies on food - do they send any information about diet - what do you think of this (pros and cons)
- What are the challenges you face to controlling your and your child’s diet?

***Questions relating to baby bottle use***

- Were you given information about baby bottle use when your child was first born?
- Can you describe what this information was, where did it come from, did it change what you did.

***Question relating to the dental visit/ hospital visit in question***

- Can you describe what/ the reasons for your child visiting the dentist/ hospital today (or for the appointment in question)?
- How did/ do you feel about the treatment?
- Did you feel you were given all the information? What were the alternatives - were there any?
- How did you (the child) feel after the treatment? (Probe still in pain )
- Did the treatment have any positive or negative impacts?
- How did you feel about how quickly you were seen?
- Has the operation or treatment affected or changed the way you see the dentist - if yes how *prompts - anxiety, happier, regular, irregular attender - how often did you see the dentist in the past - how often do you plan to see the dentist now
- Do you think you will alter any habits or behaviour after this visit to/with the dentist - if yes in what way?
- Were you given any preventative (dental) advice? > How did you feel about this advice how likely are you to follow this now

***Question relating to previous dental visits***

- Can you tell me about some of your previous dental visits for both your self and your child?
- What have you visited the dentist for - (probe describe the experience how did it make you feel did you change any behaviour after the visit)
- What has your child previously visited the dentist for (probe ask child to describe the experience how did it make you feel did you change any behaviour after the visit)
- Have you or your child ever had any anxiety about visiting the dentists - have you discussed this with the dentists - was anything done after this discussion (probe behavioral management, acclimatization)

***Questions relating to interventions***

- What services or activities could be made available to help with managing your child’s oral health and diet
- How would you want to learn about the issues and problems (probe - mobile phone message - reminders, leaflets - where would you get information from - sure start centers
- What do you do now, what have you done in the past, what would you like to do in the future, what have been the changes (why have these things changed)

# THANK YOU FOR GIVING YOUR TIME TO DISCUSS THESE ISSUES
